# Societal attention toward extinction threats: a comparison between climate change and biological invasions

**DOI:** 10.1038/s41598-020-67931-5

**Published:** 2020-07-06

**Authors:** Ivan Jarić, Céline Bellard, Franck Courchamp, Gregor Kalinkat, Yves Meinard, David L. Roberts, Ricardo A. Correia

**Affiliations:** 1 Biology Centre of the Czech Academy of Sciences, Institute of Hydrobiology, České Budějovice, Czech Republic; 2 Faculty of Science, Department of Ecosystem Biology, University of South Bohemia, České Budějovice, Czech Republic; 3 Leibniz-Institute of Freshwater Ecology and Inland Fisheries, Berlin, Germany; 4 Université Paris-Saclay, CNRS, AgroParisTech, Ecologie Systématique Evolution, 91405 Orsay, France; 5 Université Paris Dauphine, PSL Research University, CNRS, Paris, France; 6 Durrell Institute of Conservation and Ecology, School of Anthropology & Conservation, Marlowe Building, University of Kent, Canterbury, Kent UK; 7 Helsinki Lab of Interdisciplinary Conservation Science (HELICS), Department of Geosciences and Geography, University of Helsinki, Helsinki, Finland; 8 Helsinki Institute of Sustainability Science (HELSUS), University of Helsinki, Helsinki, Finland; 9 CESAM - Centre for Environmental and Marine Studies, University of Aveiro, Aveiro, Portugal; 10 Institute of Biological and Health Sciences, Federal University of Alagoas, Av. Lourival Melo Mota, Maceió, AL Brazil

**Keywords:** Conservation biology, Biodiversity, Climate-change ecology, Conservation biology, Invasive species

## Abstract

Public attention and interest in the fate of endangered species is a crucial prerequisite for effective conservation programs. Societal awareness and values will largely determine whether conservation initiatives receive necessary support and lead to adequate policy change. Using text data mining, we assessed general public attention in France, Germany and the United Kingdom toward climate change and biological invasions in relation to endangered amphibian, reptile, bird and mammal species. Our analysis revealed that public attention patterns differed among species groups and countries but was globally higher for climate change than for biological invasions. Both threats received better recognition in threatened than in non-threatened species, as well as in native species than in species from other countries and regions. We conclude that more efficient communication regarding the threat from biological invasions should be developed, and that conservation practitioners should take advantage of the existing attention toward climate change.

## Introduction

With an ongoing global biodiversity crisis, it is critical to overcome the present research-implementation gap in conservation^1,2^. Failure to halt biodiversity decline does not solely come from a lack of scientific understanding of drivers of decline, but also from a lack of societal support and action^3^. Societal awareness and values partly determine the level of support for, engagement with, and effectiveness of conservation initiatives, as the societies tend to protect only what they recognize as important^4,5^.

The emerging field of conservation culturomics provides unprecedented opportunities to understand societal attention and interests related to conservation objectives^6,7^. Culturomics aims to generate new insights into human culture through the quantitative analysis of digital data^6^. Conservation culturomics is focused on the understanding of cultural dynamics associated with conservation, and comprises diverse sources and approaches, including webpage retrieval analysis (Internet salience), assessment of web search activity, page views of digital archives such as Wikipedia, contents of social networks and online news, and images and videos posted online^8–12^. A number of studies within this field have recently focused on public attention and interest in major anthropogenic impacts on natural systems, such as climate change and biological invasions, based either on web search query data or online news media^13–22^. However, as opposed to the intensive research of general societal interest in these threats, there have been no attempts so far to assess societal attention toward extinction threats related to biodiversity, to specific species and species groups. Such studies can provide valuable insights regarding public recognition of the actual threats, and major attention gaps and biases. Furthermore, higher attention toward extinction threats faced by a particular species is likely to also contribute to perceived relevance of the species for the society and its overall visibility.

The aim of this study was to explore how much attention is directed at climate change and biological invasions in relation to different species groups. We focus on climate change and biological invasions as two key processes that affect biological diversity and are often interrelated and act synergistically^23–27^. We hypothesize that, because biological invasions are as yet not well understood regarding the invasion process and their impacts by the general public, they generate less societal attention than climate change, which can be a basic hindrance for conservation^28^. Our analysis was based on the Internet salience (i.e. the volume of Internet pages containing a particular term^29,30^) of terms referring to extinction threats in relation to multiple species. Internet salience is considered a good indicator of a species’ cultural visibility^30,31^, and it is therefore likely that this capacity also applies to species extinction threats. Furthermore, the Internet is increasingly used as a source of information^32,33^, and because most Internet content is generated by self-interest (e.g. blogs, Wikipedia, social media) or perceived interest (e.g. news outlets), more available content is likely to generate more reads and thus more attention. It is, therefore, expected that there is a strong feedback loop between content availability and public interest and attention^6^. We test the hypothesis by comparing the Internet salience of biological invasions with that of climate change, which is expected to be characterized by a much higher level of public attention. Based on webpage retrieval analysis, we assessed the Internet salience of these two threats in relation to threatened amphibian, reptile, bird and mammal species in France, Germany and United Kingdom. Our results provide novel insights into the differences in the societal attention directed at these two threats, among different countries, species and their origin, as well as in the level of attention overlap with the actual existence and severity of these threats. We address the implications of the results for conservation management and outreach planning.

## Results

**Figure 1:**
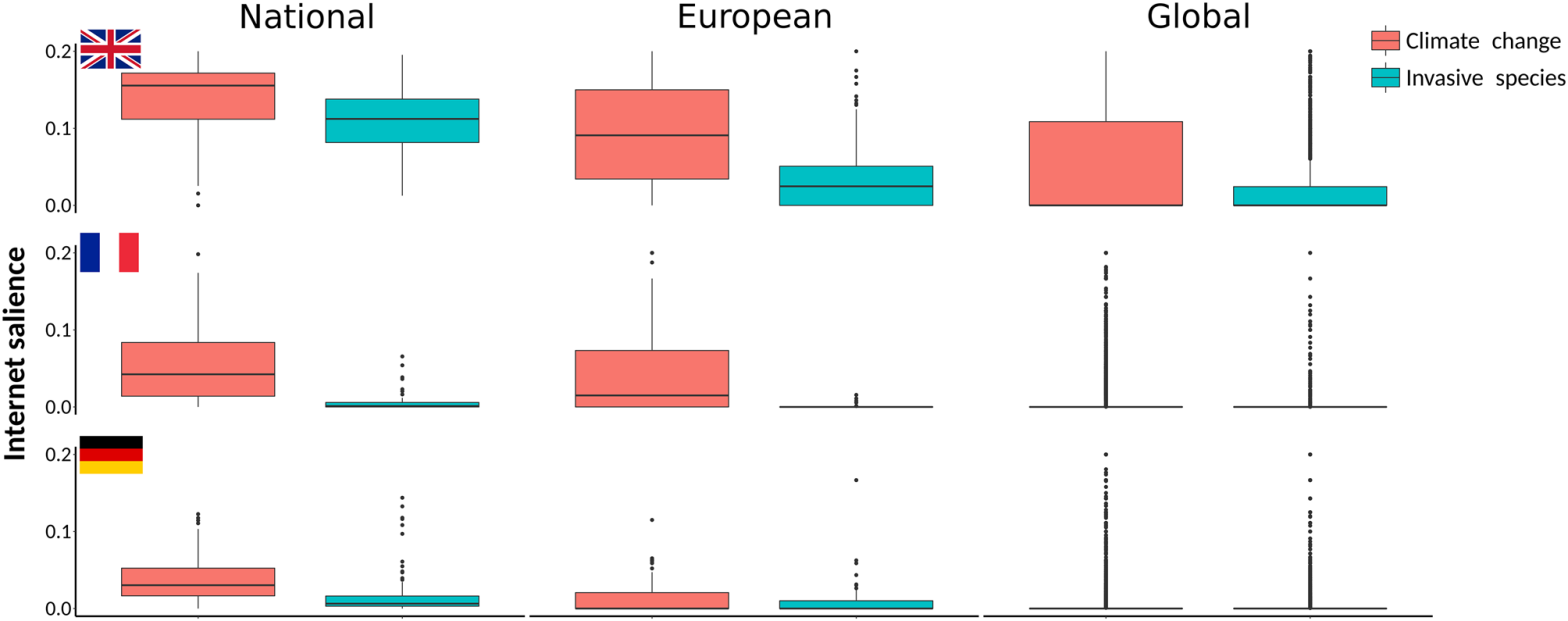
Climate change is a more salient threat on Internet than invasive alien species; both threats are also better recognized for threatened species within the country than elsewhere in Europe and in the world. Relative Internet salience of climate change and invasive alien species in relation to threatened species from United Kingdom, France and Germany, as well as to other threatened species of these groups, present elsewhere in Europe and in the world (noted in figure as national, European and global, respectively). Relative Internet salience was expressed as the mean number of webpages retrieved by Internet search within each of the countries for the scientific name of a species and the particular threat, divided by the number of webpages retrieved by searching for the scientific name only.

**Figure 2:**
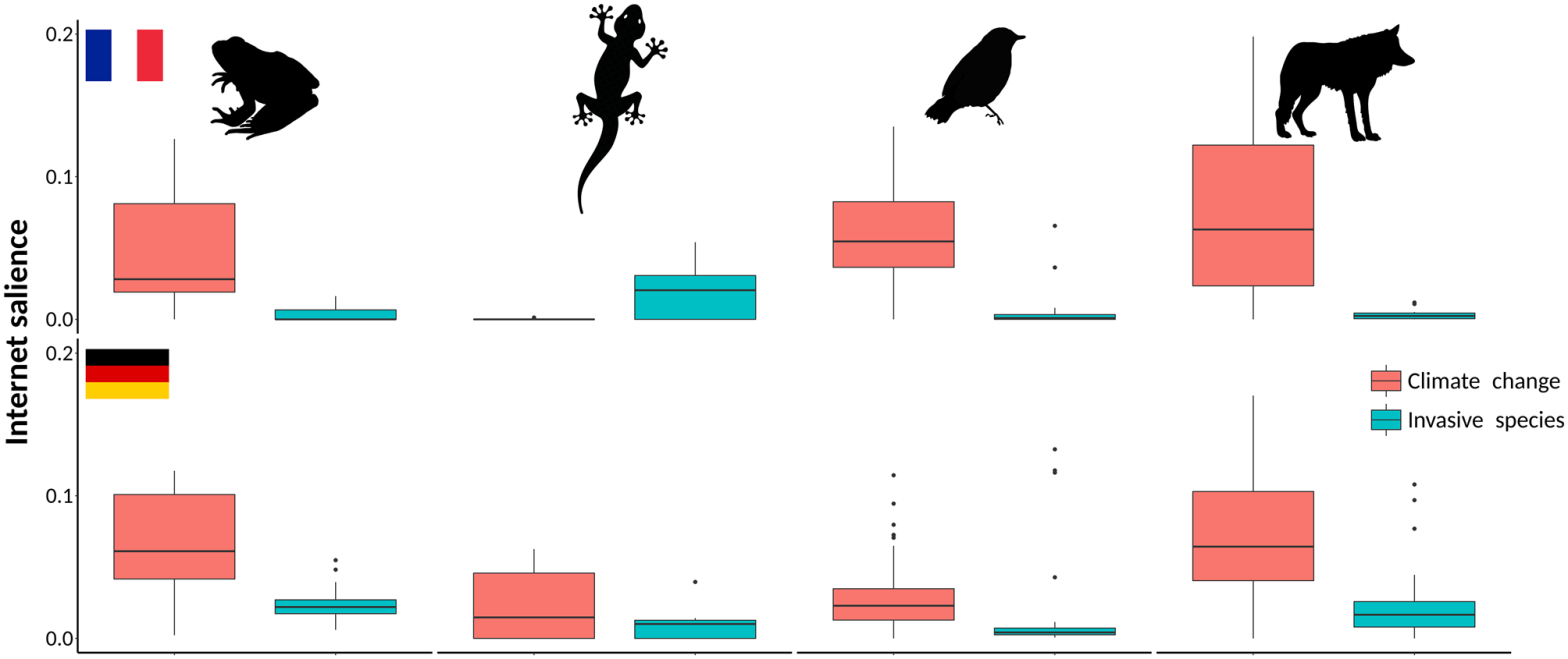
Climate change is better represented on Internet than invasive alien species in most of the studied species groups and countries. Relative Internet salience of climate change and invasive alien species in relation to threatened species from the four studied species groups (amphibians, reptiles, birds and mammals) from France and Germany. Relative Internet salience was expressed as the mean number of webpages retrieved by Internet search within each of the countries for the scientific name of a species and the particular threat, divided by the number of webpages retrieved by searching for the scientific name only. Data for United Kingdom were not presented, since the threatened species within the national red list were represented almost exclusively by birds. Images obtained from publicdomainvectors.org (CC0 1.0).

**Figure 3:**
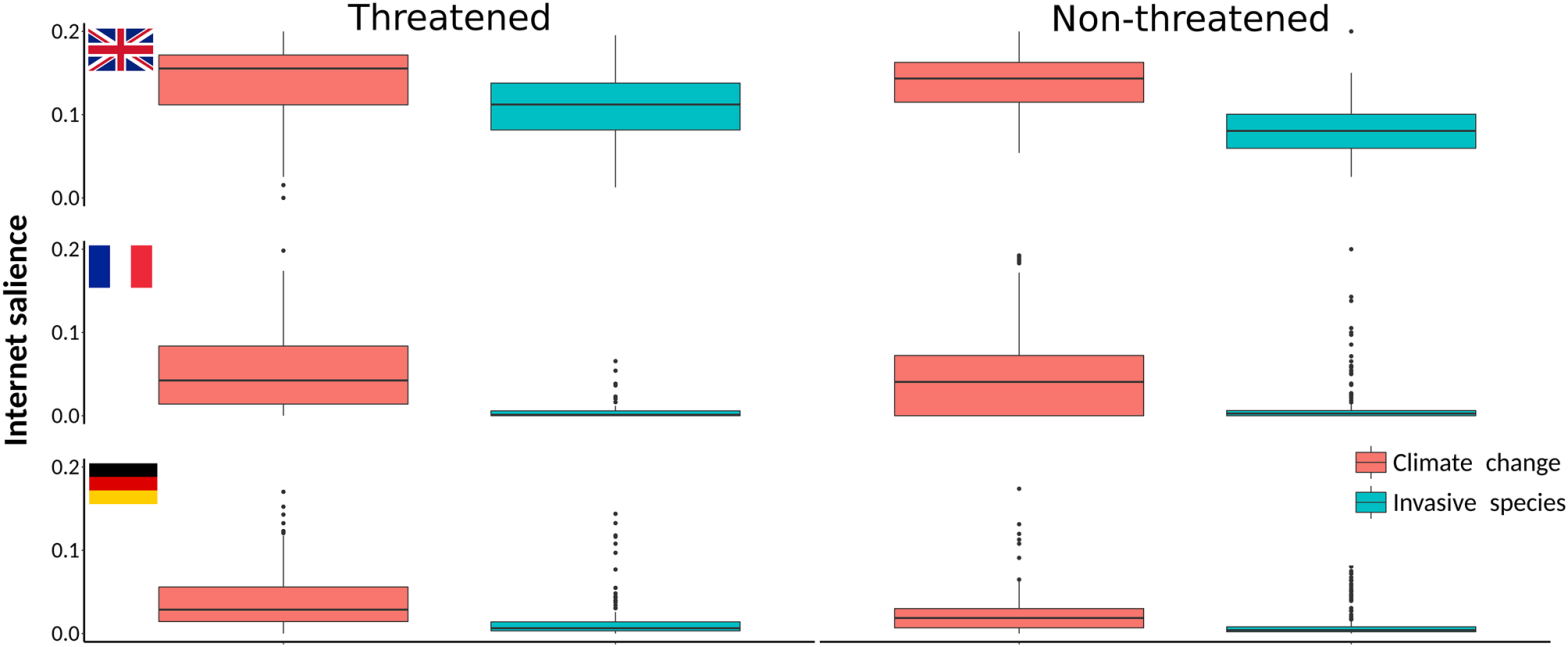
Climate change is a more salient threat on Internet than invasive alien species, especially among threatened species. Relative Internet salience of climate change and invasive alien species in relation to threatened and non-threatened species from United Kingdom, France and Germany. Relative Internet salience was expressed as the mean number of webpages retrieved by Internet search within each of the countries for the scientific name of a species and the particular threat, divided by the number of webpages retrieved by searching for the scientific name only.

**Figure 4:**
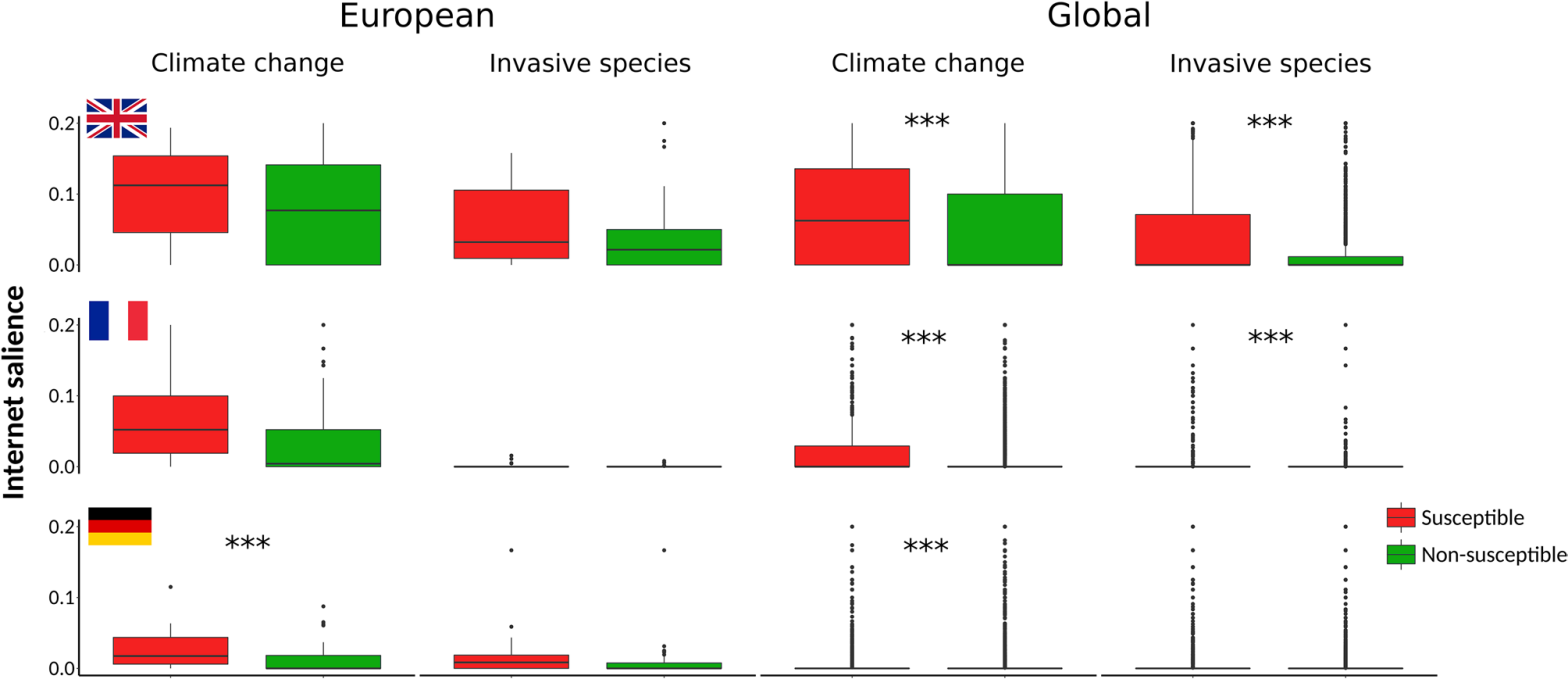
Climate change and invasive alien species are more salient on Internet for species that are considered susceptible to each of the threats. Relative Internet salience of climate change and invasive alien species in relation to threatened species within and outside of Europe, which were either classified or not classified as susceptible to the threat at issue within the IUCN Red List database^34^. Relative Internet salience was expressed as the mean number of webpages retrieved by Internet search within each of the three countries for the scientific name of a species and the particular threat, divided by the number of webpages retrieved by searching for the scientific name only. Asterisks indicate significant differences (Mann Whitney U Test with Bonferroni correction, *p* < *α*, where *α* = 0.05/12 = 0.00417).

Climate change had a significantly higher relative Internet salience than biological invasions. This was consistent among countries and species groups (Figs. 1, 2, 3, 4). Furthermore, both threats were better represented among species present in Germany, France, and United Kingdom than among those outside of each of the countries, and they were also more represented among species from Europe than among those distributed outside of Europe (Mann–Whitney U test, *p* < 0.01; Fig. 1). Climate change had a significantly higher prominence than biological invasions within amphibians, birds and mammals (Mann–Whitney U test, *p* < 0.05), while the differences in the representation of the two threats within reptiles were not significant (Fig. 2). Both threats had significantly higher Internet salience within threatened than within non-threatened species in Germany and United Kingdom (Mann–Whitney U test, *p* < 0.01), but not in France (Fig. 3). Overall Internet salience across threats, countries, and species groups is presented in Supplementary material S1.

On the other hand, the proportion of pages mentioning a certain threat that also contained names of particular species had the opposite pattern, with biological invasions being better represented than climate change (Table 1). In other words, webpages addressing climate change had less frequent mentions of particular species than those dealing with biological invasions. This was especially prominent for France (Table 1).

**Table 1:** Relationship between the Internet salience of climate change and invasive alien species when Internet search included the name of one of the threats only, and when it comprised also scientific names of threatened amphibian, reptile, bird and mammal species from United Kingdom, France and Germany.

Internet search type	Internet salience		Proportion (%, median across species and range)
	Threat + scientific name (median across species and range)	Threat	
**Climate change**			
UK	183.5 (0–552)	858,000	0.021 (0.000–0.064)
France	18.0 (0–489)	400,000	0.005 (0.000–0.122)
Germany	31.0 (0–723)	835,000	0.004 (0.000–0.087)
**Invasive alien species**			
UK	95.5 (2–354)	192,000	0.050 (0.001–0.184)
France	1.0 (0–63)	1,450	0.069 (0.000–4.345)
Germany	6.0 (0–384)	9,060	0.066 (0.000–4.238)

Species that were recognized as susceptible to either climate change or biological invasions within the IUCN Red List database^34^ overall had a better online representation of the given threat (i.e., such species were more often mentioned together with the threat on the same webpage) than those that were not recognized as susceptible. This indicates that the representation of threats on the Internet corresponds well to expert identification. While the Internet salience of threats was positively, but weakly correlated with threat severity in species impacted by a given threat, this was consistent across countries and threats (Table 2). Such results indicate the potential presence of a pattern, albeit a weak one, where species that are more strongly impacted by either biological invasions or climate change were characterized by a better representation of that threat on the Internet (Table 2).

**Table 2:** Coefficients of correlation (Spearman's non-parametric correlation test) between the relative Internet salience of climate change and invasive alien species and the assigned threat severity within the IUCN Red List database^34^.

	UK	France	Germany
Climate change	0.13**	0.12**	0.10*
Invasive alien species	0.16**	0.18**	0.11**

Dataset comprises threatened species from the four studied taxon groups from United Kingdom, France and Germany, classified as susceptible to the threat at issue within this database.

*p < 0.05; **p < 0.01.

## Discussion

Our results revealed substantial differences in the Internet salience of the two studied threats, with a considerably higher prominence of climate change as a threat in assessed species groups than biological invasions. Such results were expected and intuitive. Humans have moved species across the world for hundreds of years, but the sharp changes in global climate in response to human activity have only been occurring over the past few decades^17,35^. This is also supported by the dominance and an increasing trend in climate change frequency in Internet search queries and media, as opposed to biological invasions and other conservation topics that were characterized by considerably lower and often declining trends^13,18–21^. One of the reasons for a higher visibility of climate change is that it is experienced directly by the public, with easily perceived global effects and economic damages, while biological invasions and most of the other threats are perceived as more local, context-dependent and indirect impacts^20^. Research related to climate change is a larger and more multidisciplinary field than invasion science^36^, with multiple scientific disciplines and a large number of researchers involved. This consequently translates into more intense and effective science outreach. Visibility of and interest in climate change is also further enhanced by the presence of an intergovernmental panel of scientists on climate change (IPCC), but probably also by ongoing debates about its causes, effects and very existence. On the other hand, activities of the Intergovernmental Science-Policy Platform on Biodiversity and Ecosystem Services (IPBES), including the recent publication of the Global Assessment Report on Biodiversity and Ecosystem Services^37^ and the establishment of a special working group for invasive alien species, are likely to help improve communication about biological invasions. Society needs to be aware and recognize the reality of a certain threat, as well as that it affects particular species, before it can become interested and invested in the related management issues. In this respect, attention toward the impacts of these two threats on different species is also heavily affected by the prominence of climate change scepticism and invasive species denialism^35,38–40^, as well as by societal perception of particular invasive alien species and their impacts^41,42^.

Climate change was however much less associated with species than biological invasions (Table 1), and one of the reasons behind this pattern might be the way these threats are perceived and addressed in management. For managers in the field, acting directly on climate change is in most cases impossible, and while climate change is relevant to local management, it is less so in terms of individual species. Climate change effects are also associated with other disciplines beside those dealing with species extinctions, and other issues beyond the concern for particular species, which are widely communicated to the general public. Consequently, managers may less frequently mention climate change in actions to be implemented that are targeting particular species. Rare exceptions are species that could be artificially translocated by humans to more suitable climate zones^43,44^, as well as those considered for measures related to genetic management, such as selection of resistant genotypes and genetic modifications. On the other hand, management of biological invasions often takes into consideration species that are threatened by a particular harmful invader, and such species are usually also explicitly addressed in the management^45^.

The wider recognition of climate change as a biodiversity threat compared to invasive species motivates the idea that more should be done to foster awareness of the less known threat. This is especially relevant considering that biological invasions were thus far a comparable threat to climate change in terms of species extinctions. In fact, invasive species have apparently led so far to a larger number of extinctions than climate change^24,46^. There are examples of invasive species that have already led to local and regional extinctions in Europe^47,48^, with hundreds of invasive species listed in Europe with manifested impacts on native biodiversity, mainly originating from America and Asia^49^. While both threats are driven by human behavior and while climate change may be particularly a global issue, much of the actions by the society are local, which makes the two threats comparable.

Both threats were characterized by notable differences among countries, species groups and their origin. Either threat had much higher coverage related to species native for a given country than to those from other countries and regions. People are more knowledgeable of their immediate surroundings, and the species familiarity and the range proximity to or overlap with developed nations are recognized drivers of both societal and scientific taxonomic attention^10,31^. This is further strengthened by regional differences and gaps in knowledge and information about these threats, due to strong spatial research biases^50,51^. Regional and country-level differences in societal taxonomic attention and interest in conservation issues have been also observed in other studies^8,15^. The United Kingdom showed a particular pattern related to invasive alien species compared to the two other countries, with considerably higher online association of species with the threat. Many interpretations could be found for the higher internet salience in the United Kingdom related to biological invasions, such as the Commonwealth relationships and the effect of insularity. Islands are recognized as considerably more vulnerable to invasions^52^. However, while the United Kingdom represents an island, substantial differences between the United Kingdom territory and mainland Europe in the invasion risks and vulnerability are unlikely, due to recent glaciation and the presence of a land bridge between the two regions^53,54^. On the other hand, the insularity can strengthen negative attitudes of the society towards biological invasions, as is the case for example in New Zealand^55^. The observed differences among countries are likely to be driven by a complex set of factors, which warrants further studies.

The highest overall Internet salience of the two threats was observed in mammals, followed by birds, amphibians and reptiles. The same pattern in societal attention towards the four species groups was observed previously in Internet search frequency^5,33^, social media use^56^, Twitter activity and Wikipedia page visits following screening of natural history movies^57^, and the choice of species on covers of conservation magazines^58^. High Internet salience of mammals is largely driven by their charisma and the resulting increased societal attention and communication. Mammals are considered to be the most charismatic species group^59,60^, driven by a complex set of traits including body size, phylogenetic proximity to humans, anthropomorphism, unique morphology, elaborate coloration patterns, neotenic features, complex behavior and sociality^4,5,61–63^, as well as culturally embedded symbolic values^63,64^. Other drivers of Internet salience of threats among the four species groups include their distribution and abundance within each country, their threat status, ecology, and accessibility for research^10^. Disentangling effects of such a range of different drivers would however require detailed analyses that are beyond the scope of the present study.

Two potentially encouraging results of the study are higher societal interest in the two threats in threatened than within non-threatened species, as well as that the IUCN Red List listing of threats and their severity in different species were reflected in threat coverage among species (i.e. by a higher Internet salience of a threat in species classified as affected by the given threat, as well as by increasing Internet salience with threat severity). This might suggest a good level of societal attention toward threats and proper coverage of scientific work and available knowledge, supporting recent findings that suggest a high level of overlap between scientific and societal taxonomic attentions^10^. However, while knowledge and awareness of threats is a necessary condition for actual conservation engagement, we should bear in mind that they alone are insufficient to guarantee engagement.

It is important to note some potential limitations of the study, that call for due caution when interpreting results. Use of vernacular names tends to be much more prominent in media than scientific names, which means that by focusing analyses only on scientific names an important part of public discussions on threats related to particular species might be missed. This is however unavoidable in order to ensure unbiased data sampling, considering numerous problems associated with the use of vernacular names to identify species in the literature^29,30,65^. Assessments of species coverage in news and social media based on scientific names have to be interpreted with due caution, especially when the focus is on text contents or absolute results. On the other hand, their use to assess relative coverage, for temporal trends or comparisons among species groups, was validated as reliable^9,29,30^. Furthermore, species characteristics and charisma can affect the style and tone of the language used by media outlets^55,63,66^. Conservation management is frequently associated with conflicts, with reporting often resorting to emotive or militaristic language and war-like metaphors to advocate particular perspectives^66,67^. The choice of language and the usage of particular phrases such as euphemisms and buzzwords have to be taken into consideration when applying language processing methods^68,69^. Additionally, use of phrases such as "invasive species" and "climate change" can sometimes be ambiguous and perceived by the public with a different meaning than the one established in science, not necessarily reflecting perception of these threats as conservation issues. For example, people sometimes tend to refer to native species whose populations are on the rise, such as roe deer (*Capreolus capreolus*) or wild boar (*Sus scrofa*), as “invasive species”. However, even though it was not possible to monitor the effects of such ambiguities within the assessed dataset, they are probably too scarce to significantly affect results. Furthermore, the potential problem of global unevenness of the public access and use of Internet was avoided here by focusing on European countries, where most of the population are active Internet users. Finally, use of web search engines also requires caution, because the results returned may vary with time, both due to changes in search engine algorithms (e.g. personalization algorithms, webpage indexation) and temporal instability of web pages. Nevertheless, focusing our analysis on relative differences instead of absolute values ensures that the overall patterns should be robust to these variations.

One part of our analysis included comparisons of the species vulnerability to threats based on the IUCN Red List threat classification with Internet salience of threats. Reliability of the IUCN Red List threat classification was sometimes questioned, especially regarding climate change threat assignment^70^. Nevertheless, recent studies have shown good performance of IUCN Red List threat assignment^71,72^, while a set of objective threat assignment criteria established by IUCN and a wide pool of expert assessors involved in the assessment should ensure that the database represents the best available knowledge ^73^.

The observed disparity in the online coverage of climate change and biological invasions supports recent claims that general attention toward biological invasions seems to be limited^28^. In addition, the low Internet salience of climate change and biological invasions in relation to individual threatened species, when compared to the general salience of the two threats (Table 1), also indicated that species-specific conservation concerns represent a small proportion of the overall Internet content. Yet, Internet coverage of the two threats also revealed the presence of two general communication models: climate change is a well-recognized threat, but addressed in a more general way, mostly not associated with specific species; on the other hand, biological invasions are less recognized, but they are more often associated with particular impacted species. There is a need to devise more efficient communication and outreach approaches regarding the threat from biological invasions, especially since our results may imply that a lack of attention is in part driven by the media. A set of tools from climate change communication that were advocated by Legagneux et al.^20^ for improved communication on biological invasions, including the use of metaphors, iconic species and mobilizing information, could be a step in the right direction.

## Methods

We compiled a dataset with species scientific names (including synonyms), taxonomy, Red List categories, threat classification and threat severity (i.e., the overall decline caused by the threat, expressed as the magnitude of population decline over 10 years or three generations) based on data from the IUCN Red List database^34^. The dataset comprised 5,988 threatened mammal, bird, reptile and amphibian species (i.e. classified as Vulnerable (VU), Endangered (EN) or Critically Endangered (CR)). In addition to the global red list, national red lists of Germany, France and United Kingdom, including both nationally threatened and non-threatened species from the four taxon groups (excluding species from overseas territories), were also downloaded from the National Red List database (https://www.nationalredlist.org—downloaded on 14 December 2017). Threatened species in Germany were considered to be those with the national status being either “Declining Species”, “Possibly Endangered”, “Endangered”, “Critically Endangered”, “Extremely Rare”, or “Threatened with Extinction”; in United Kingdom, it was either "Amber" or "Red" national status, and "Vulnerable", "Endangered" or "Critically Endangered" in France. Since one of the objects of the study was to compare Internet salience of threats for threatened and non-threatened species from each country, and the German national red list comprised exclusively threatened species, a list of non-threatened species from Germany was obtained from the global IUCN Red List database [i.e., species classified as Least Concern (LC) or Near Threatened (NT)]^34^. List of non-threatened species from United Kingdom included those with the national status "Green", and LC or NT as a national status those from France.

Internet salience data collection was performed during March 2018, in line with the approach proposed by Jarić et al.^29^ and Correia et al.^30^. Search was conducted using scientific species names, including synonyms^9^. Scientific names represent a reliable proxy and preferable alternative to vernacular names, due to a strong and culturally independent association between their representation in digital corpora^29,30^. Use of vernacular names is problematic and prone to biases, due to their tendency to have loose and multiple meanings, differing vernacular names among different languages and multiple names within the same language, species-unrelated uses of vernacular names (e.g. names of companies, sport teams, commercial products, toponyms, personal names, etc.), as well as a lack of vernacular names in certain species groups^29,30^. Scientific names allow researchers to conduct standardized assessments across different language groups, effectively controlling for many of the potential biases and avoiding above described problems related to vernacular names^29,30^. Furthermore, the aim of such analyses is not to identify all mentions of a species on the Internet, but rather to reliably detect patterns based on a robust sample of webpages. Google’s Custom Search Engine API was used to search websites from each of the three countries for pages mentioning scientific species names, terms representing the two threats (i.e., climate change and invasive species, respectively), and their combination. Species name search included both recognized scientific names and synonyms, placed in parentheses within a same search query (i.e., [“*species name*” OR “*synonym #1*” OR “*synonym #2*” OR…]), to avoid negative effect of taxonomic synonyms on search accuracy^9^. Searches for the two threats were performed with the commonly used terms in each country (Table 3). Although the focus of the study were species and threats in the three countries within Europe, Google’s Custom Search Engine API also covered data from their overseas territories. Nevertheless, online material originating from those regions is unlikely to substantially affect our results, mainly due to the small proportion of the human population of overseas territories (~ 4% for France, < 1% for United Kingdom).

**Table 3:** Terms used for Internet search for the two threats for each country.

	Climate change	Invasive species
Germany	[“Klimawandel” OR “Erdewärmung” OR “globale Erwärmung”]	[“invasive Arten”]
France	[“changement climatique” OR “dérèglement climatique” OR “réchauffement climatique” OR “réchauffement planétaire” OR “réchauffement de la planète” OR “réchauffement de la terre”]	[“espèces envahissantes” OR “espèces invasives”]
UK	[“climate change” OR “global warming”]	[“invasive species”]

Data analysis was based on the relative Internet salience of the two threats in relation to each of the species. Relative Internet salience was expressed as the proportion of webpages that include particular species name and the given threat to all webpages that mention that species ([“*species name”* AND “*threat name*”] / [“*species name*”]). Analysis included comparisons of relative Internet salience between the two threats, among national, European and global threatened species pools, between threatened and non-threatened species, among the four species groups, among the three countries, and between species classified as affected by the threat at issue within the IUCN Red List database and those that are not. We also assessed relative Internet salience of the studied species in relation to the two threats, by expressing the Internet salience as the proportion of webpages that includes species name and the given threat to all webpages that mention that threat ([“*species n*ame” AND “*threat n*ame”] / [“*threat n*ame”]).

Since data were not normally distributed (Kolmogorov–Smirnov test, *p* < 0.001), statistical analyses were performed using nonparametric tests. Differences in Internet salience between groups were assessed using the Mann–Whitney U test with Bonferroni correction, with the following comparisons: 1) between the two threats within each country, 2) between threatened and non-threatened species for each threat and country, 3) among the three countries for each threat, 4) among the four species groups for each threat and country, 5) among national, European and global species pools for each threat and country, and 6) between species classified within IUCN Red List as affected by each threat and those that are not for each country. Groups comprised only threatened species in all comparisons, except for (2), with all species groups pooled together, except for (4). The relationship between relative Internet salience and the severity of each threat^34^ was assessed using the Spearman’s Rank test.

## Supplementary information

Supplementary information
