# Supplementary material for: Societal attention toward extinction threats: a comparison between climate change and biological invasions
Source: Sci Rep. 2020 Jul 6;10:11085. doi: 10.1038/s41598-020-67931-5 (PMC7338409; doi:10.1038/s41598-020-67931-5)
Supplement: Supplementary file 1 — Supplementary information [file 41598_2020_67931_MOESM1_ESM.doc]

**Electronic Supplementary Material S1**

**Societal attention toward extinction threats: A comparison between climate change and biological invasions**

Authors: Ivan Jarić^*^, Céline Bellard, Franck Courchamp, Gregor Kalinkat, Yves Meinard, David L. Roberts and Ricardo A. Correia

^*^ Corresponding author: Biology Centre of the Czech Academy of Sciences, Institute of Hydrobiology, Na Sádkách 702/7, 370 05 České Budějovice, Czech Republic. E-mail addresses: ivan.jaric@hbu.cas.cz

**Table S1.** Relationship between the Internet salience of climate change and invasive alien species when Internet search included scientific names of amphibian, reptile, bird and mammal species from national red lists United Kingdom, France and Germany (including species listed as both threatened and non-threatened), and when it comprised also the name of one of the threats.

| Internet search type | | | Internet salience (median across species and range in parentheses) | | Proportion (median across species and range in parentheses) |
| --- | --- | --- | --- | --- | --- |
|  |  |  | Threat + scientific name | Scientific name |  |
| Climate change | UK^*^ | Amphibians | 511.0 | 1390.0 | 0.368 |
|  |  | Reptiles | 315.0 | 1520.0 | 0.207 |
|  |  | Birds | 182.5 (0 - 552) | 802.5 (10 - 3880) | 0.202 (0.000 - 0.508) |
|  |  | Mammals | / | / | / |
|  |  | All groups | 183.5 (0 - 552) | 812.5 (10 - 3880) | 0.204 (0.000 - 0.508) |
|  | France | Amphibians | 3.0 (0 - 182) | 273.0 (0 - 2990) | 0.032 (0.000 - 0.241) |
|  |  | Reptiles | 0.0 (0 - 307) | 52.0 (0 - 3130) | 0.000 (0.000 - 0.134) |
|  |  | Birds | 25.0 (0 - 275) | 470.0 (0 - 11700) | 0.050 (0.000 - 0.172) |
|  |  | Mammals | 27.0 (0 - 489) | 424.0 (0 - 25800) | 0.050 (0.000 - 0.269) |
|  |  | All groups | 18.0 (0 - 489) | 387.0 (0 - 25800) | 0.045 (0.000 - 0.269) |
|  | Germany | Amphibians | 7.0 (0 - 235) | 341.0 (0 - 2330) | 0.022 (0.000 - 0.118) |
|  |  | Reptiles | 0.5 (0 - 114) | 87.0 (0 - 2040) | 0.010 (0.000 - 0.063) |
|  |  | Birds | 36.0 (0 - 617) | 1660.0 (0 - 61900) | 0.021 (0.000 - 0.114) |
|  |  | Mammals | 17.0 (0 - 723) | 435.0 (0 - 23700) | 0.044 (0.000 - 1.000) |
|  |  | All groups | 31.0 (0 - 723) | 1340.0 (0 - 61900) | 0.024 (0.000 - 1.000) |
| Invasive alien species | UK^*^ | Amphibians | 354.0 | 1390.0 | 0.255 |
|  |  | Reptiles | 185.0 | 1520.0 | 0.122 |
|  |  | Birds | 94.5 (2 - 296) | 802.5 (10 - 3880) | 0.107 (0.013 - 0.848) |
|  |  | Mammals | / | / | / |
|  |  | All groups | 95.5 (2 - 354) | 812.5 (10 - 3880) | 0.107 (0.013 - 0.848) |
|  | France | Amphibians | 2.0 (0 - 63) | 273.0 (0 - 2990) | 0.006 (0.000 - 0.071) |
|  |  | Reptiles | 1.0 (0 - 16) | 52.0 (0 - 3130) | 0.004 (0.000 - 0.300) |
|  |  | Birds | 1.0 (0 - 12) | 470.0 (0 - 11700) | 0.001 (0.000 - 0.066) |
|  |  | Mammals | 2.0 (0 - 44) | 424.0 (0 - 25800) | 0.003 (0.000 - 1.000) |
|  |  | All groups | 1.0 (0 - 63) | 387.0 (0 - 25800) | 0.002 (0.000 - 1.000) |
|  | Germany | Amphibians | 15.0 (0 - 97) | 341.0 (0 - 2330) | 0.039 (0.006 - 0.083) |
|  |  | Reptiles | 2.0 (0 - 50) | 87.0 (0 - 2040) | 0.011 (0.000 - 0.052) |
|  |  | Birds | 6.0 (0 - 384) | 1660.0 (0 - 61900) | 0.004 (0.000 - 0.133) |
|  |  | Mammals | 6.0 (0 - 308) | 435.0 (0 - 23700) | 0.017 (0.000 - 1.000) |
|  |  | All groups | 6.0 (0 - 384) | 1340.0 (0 - 61900) | 0.005 (0.000 - 1.000) |

* UK dataset comprised no mammals, and only a single amphibian and reptile species
